# Effect of areca nut extracts on growth performance, slaughtering performance, and meat quality of broiler chickens

**DOI:** 10.3389/fvets.2025.1579415

**Published:** 2025-04-28

**Authors:** Juan Xu, Mengyao Li, Rong Li, Zhen Zhang, Ying Ma, Ran Tao, Lirui Zou, Ji Wang, Lixin Wen, Rongfang Li

**Affiliations:** ^1^Hunan Engineering Research Center of Livestock and Poultry Health Care, Colleges of Veterinary Medicine, Hunan Agricultural University, Changsha, China; ^2^Changsha Lvye Biotechnology Co., Ltd, Changsha, China

**Keywords:** areca nut extract, broiler, growth performance, slaughtering performance, meat quality

## Abstract

**Introduction:**

Since the comprehensive ban on the addition of antibiotics to livestock and poultry feeds in China, the search for safe and natural antibiotic substitutes has become a hot spot in the animal breeding industry. Areca catechu L (AN), known as the leader among the four southern medicinal herbs, possesses functions such as insecticidal, antibacterial, antiinflammatory, promoting gastrointestinal motility and preventing Alzheimer's disease. Nevertheless, ANE is rarely used as a feed additive in AA broilers, and its specific role remains unclear. This study was conducted to investigate the effects of different levels of areca nut extracts (ANE) on growth performance, slaughter performance and meat quality of AA broiler chickens.

**Methods:**

128 one-day-old Arbor Acres broilers were randomly divided into eight groups of 16 birds each, housed in three cages with 5-6 birds per cage, with or without ANE supplementation (0, 100, 150, 200, 250, 300, 350, and 400 mg/kg, respectively).

**Results:**

The entire experiment duration was 49 days. Adding 100 and 200 mg/kg ANE to the diet could significantly increase the body weight of broilers at 21 days of age (*P* ≤ 0.001), and significantly reduce the feed-to-weight ratio from 1 to 21 days of age (*P* ≤ 0.001). The diet supplemented with 200 mg/kg ANE could significantly increase the average body weight at 49 days of age (*P* = 0.001). Compared with the control group, the addition of different dosages of ANE in the feed could improve the pH_45min_, yellowness, and shear force (*P* ≤ 0.043) of the pectoral and leg muscles of broilers. Additionally, the contents of crude fat and crude protein, which are nutritional components in the pectoral and leg muscles of the ANE-supplemented groups, were to an extent higher than those of the control group (*P* ≤ 0.036). The addition of different levels of ANE in the diet significantly raised the expression levels of muscle development-related genes, including *Myf5, Myf6, MyoD1, IGF-1*, and *IGF-2* (*P* ≤ 0.032).

**Discussion:**

In conclusion, An appropriate amount of ANE in the diet has been demonstrated to boost the growth performance and meat quality of broilers, facilitate muscle development, and has no remarkable influence on slaughter performance. the ideal dosage for broilers is 100–200 mg/kg ANE. The findings of this study offer valuable insights into the potential benefits of ANE in poultry production, and provide a basis for further research into the development of ANE as a new feed additive.

## 1 Introduction

Antibiotics were formerly extensively utilized in livestock production. Nevertheless, due to the chronic addition of antibiotics leading to drug residues, bacterial resistance, and so on (1, 2), many countries have proscribed their employment. This ban represents a major challenge for the entire livestock industry and a significant threat to animal welfare. Therefore, there is an urgent need to discover natural, environmentally friendly and safe feed additives.

Many researchers have come to pay attention to feed additives such as natural plant extracts, intestinal health agents, and digestive enzymes (3, 4). These additives cannot merely improve the immunity and production performance of animals (5), but also alleviate the issue of drug residues in food. Among these, natural plant extracts are one of the prevalent feed additives.

*Areca catechu* L. is a genus of *A. catechu* belonging to monocotyledonous palmae, distributed in the tropical and subtropical regions in southern China and other South Asian and Southeast Asian countries (6, 7). *A. catechu*, the dried seed of Areca palm fruit, is not only one of the commonly used traditional Chinese medicinal materials, but also a traditional leisure food in many regions, and over 600 million people globally frequently chew Areca nut (AN) (6). However, long-term chewing of AN can led to oral submucous fibrosis and, eventually, oral cancer. Hence, the safety issue of AN consumption has aroused widespread concern in society. Whereas, as a traditional medicinal plant (with a medicinal history of more than 2,000 years in China) (8), the physiological activity and medicinal value of AN have been constantly overlooked by the public. Researches have verified that AN possesses a variety of bioactive substances (9), such as alkaloids, phenols, flavonoids, tannins, triterpenes, fatty acids and other functional compounds (10), with multiple physiological activities such as antioxidation, alleviation of fatigue, improvement of gastrointestinal function, treatment of diabetes, restoration of the nervous system, and prevention of cancer. Its main active ingredient, arecoline, has been shown to have antibacterial and anti-inflammatory effects (11), and to stimulate gastrointestinal peristalsis (12). Some studies have found that areca nut extracts (ANE) can be used to eliminate parasites (13), treat gastrointestinal inflammation in dyspepsia (14), and improve the growth performance and intestinal microbiota in Wenchang chickens (15). Nevertheless, ANE is rarely used as a feed additive in AA broilers, and its specific role remains unclear.

Here, this study aims to comprehensively assess the effects of different levels of ANE on growth performance, slaughter performance, meat quality, and muscle nutritional components of AA broilers, with the anticipation of providing referential significance for its utilization as a feed additive.

## 2 Materials and methods

### 2.1 Preparation of ANE

The ANE is provided by Changsha Lvye Biotechnology Co., Ltd. (Changsha, China). The preparation process of the extract was as follows: first, mold-contaminated areca nuts and impurities such as dust were removed through manual inspection and sieving. The cleaned nuts were then pulverized and subjected to percolation (with a solvent-to-material ratio of 8:1), followed by concentration and drying to obtain a solid block of ANE. Finally, the solid extract was ground into powder using an extract pulverization unit, then stored at 4°C to maintain its bioactivity until further analysis and incorporation into the experimental diets. The mass of the lyophilized extract was precisely measured to ensure accurate dosage calculations for feed formulation. The active component polyphenols were determined by folin-phenol colorimetry at a content of 10.73%, and arecoline were quantified by high-performance liquid chromatography (HPLC) at a content of 3.07% (16).

### 2.2 Animals, experimental design, and diets

The experimental procedures complied with the Animal Care and Use Guidelines of China, and were approved by the Animal Care Committee of Hunan Agricultural University (No. 2021090). A total of 128 one-day-old Arbor Acres broilers with an initial body weight of 44.0 ± 1.0 g were randomly divided into 8 groups, with 16 chickens in each group. The chickens were housed in three cages per group, with 5–6 chickens per cage. The cage dimensions for 5 chickens were 0.82 m wide × 1.22 m long × 0.82 m high, and for 6 chickens, the dimensions were 0.82 m wide × 1.46 m long × 0.82 m high. The stocking density was 0.164 m3 per chicken. One group was fed with basic diet, while the remaining testing groups were fed with a basic diet containing 100, 150, 200, 250, 300, 350, and 400 mg/kg ANE. The experimental diets were provided in powdered form and formulated under commission by Changsha Lvye Biotechnology Co., Ltd., China. The feeding experiment was conducted over 49 days, encompassing both the starter stage (1–21 days) and the grower stage (22–49 days). The chicks received a corn-soybean meal diet formulated to meet or exceed the recommended nutrient requirements of broilers. The feed formulation was formulated according to the Poultry Nutrient Requirements (17). Table 1 presents the composition and nutrient levels of the experimental diets.

**Table 1:** Ingredient and nutrient levels of the basal diet (%, as-is basis).

**Items**	**Contents**	
	**Starter (1–21 d)**	**Grower (22–49 d)**
Corn	57	61.5
Soybean meal	31	24.5
Fish meal	4	5
Oil	4	5
CaHPO_4_	1.47	1.37
Limestone	0.9	1
LL-Lysine HCL, %	0.25	0.3
DL-Methionine, %	0.2	0.15
Threonine, %	0.15	0.15
Choline chloride (50%)	0.2	0.2
NaCl	0.3	0.3
Multi vitamin-mineral	0.03	0.03
Premix ^a^	0.5	0.5
Total	100.00	100.00
**Nutritional levels, %**		
Metabolizable energy (MJ/kg)	12.47	12.43
Crude protein, %	21.2	19.4
Crude fat, %	2.8	2.9
Crude fiber, %	2.7	2.3
Total phosphorus, %	0.3	0.3
Availablephosphorus, %	0.16	0.16
Calcium, %	0.21	0.29
Lysine, %	1.15	1.03
Methionine, %	0.35	0.34
Methionine + cystine, %	0.68	0.65
Threonine, %	0.73	0.72
Tryptophan, %	0.23	0.20

^a^Provided per kilogram of complete diet: vitamin A 9, 900 IU, vitamin D 33, 600 IU, vitamin E 24 IU, vitamin K 2.4 mg, vitamin B1 1.8 mg, vitamin B2 7.5 mg, vitamin B6 3 mg, vitamin B12 0.018 mg, D-biotin 0.09 mg, vitamin B9 1.2 mg, Nicotinamide 36 mg, D-pantothenic acid 9.6 mg.

All the chickens were housed in an environmentally controlled room. During the experimental period, the chickens had free access to food and water. Room temperature was set at 33°C for the first week and reduced by 1°C every 2 days until the birds reached 22°C (18). Light control: 24 h light for 1–3 days, 20~22 h light for 4–7 days and 12 h light on every day thereafter.

### 2.3 Growth performance

The growth performance of broilers was evaluated by measuring body weight (BW), average daily gain (ADG), average daily feed intake (ADFI), and feed-to-weight ratio (F/G). All birds in each group were individually weighed weekly until slaughter. ADG was calculated for sequential intervals (1–7, 7–14, 14–21, 21–28, 28–35, 35–42, and 42–49 days) and cumulative phases (1–21, 21–49, and 1–49 days). Daily health monitoring was implemented, and deceased birds were promptly removed. Feed intake was monitored daily, with residual feed quantified weekly to determine precise consumption (excluding deceased individuals). Subsequently, ADG and ADFI were adjusted using mortality-adjusted live-day correction factors based on the number of deceased birds and their survival duration (19). The established formulas are as follows (20):


$$\begin{array}{l} \text{ ADG}=\left(\text{final body weight}-\text{initial body weight}\right)/\\ \text{ number of trial days};\\ \text{ ADFI}=\left(\text{feed intake}-\text{residual feed intake}\right)/\\ \;(\text{test days }\times \text{ number of animals});\\ \text{ F}/\text{G}=\text{ADFI}/\text{ADG};\\ \text{ Body weight }\left(\text{BW}\right)=\text{total weight per cage}/\\ \text{ number of chickens per cage}. \end{array}$$

### 2.4 Serum assay

At the end of the experiment, all chickens were deprived of feed for 12 h, but water was offered *ad libitum*. Subsequently, blood samples of approximately 8 mL (21) were withdrawn from the wing vein of each chicken and left for 2 h at room temperature, then centrifuged at 3,000 rpm for 10 min and deposited at −20°C until analysis. The serum concentrations of alanine aminotransferase (ALT), aspartate aminotransferase (AST), total protein (TP), albumin (ALB), triglyceride (TG), cholesterol (TC), creatinine (CREA), urea (UREA), uric acid (UA), high-density lipoprotein (HDL-C) and low-density lipoprotein (LDL-C) were measured using a Mindray Animal Automatic Blood Biochemical Analyzer (BS-240VET) (22, 23).

### 2.5 Slaughter performance

After blood sampling, the chickens (all chickens from each replicate) were killed by cervical dislocation. The slaughter weight, half-empty weight, full-empty weight, pectoral muscle weight, leg muscle weight, abdominal fat weight, and skin and subcutaneous fat weight were determined in accordance with the Poultry Production Performance Terminology and Measurement Calculation Method (NY/T 823-2020). The slaughter rate, half-empty weight rate, full-empty weight rate, pectoral muscle rate, leg muscle rate, abdominal fat rate and skin and subcutaneous fat rate of each chicken were calculated based on the weight (24).

### 2.6 Meat color and PH measurement

Meat color was ascertained using a CR-400 colorimeter. Twenty grams of each chicken was employed to establish the Opto value 45 min after slaughter. Each meat sample was measured five times, and the mean value was obtained. The pH of intrathoracic muscles was gauged 45 min after slaughter (pH_45min_) using a carcass meat quality pH-Star meter (Matthaus, Germany). Each meat sample was measured five times, and the average was taken. The samples were then placed in sealed bags, labeled, and stored in a 4°C refrigerator for 24 h. The pH was measured again after 24 h (pH_24h_) (25, 26).

### 2.7 Muscle cooking loss measurement

To measure cooking loss, muscle samples were dried with paper and weighed 45 min after slaughter (W1). The samples were placed in a plastic bag and cooked in a hot water bath until the temperature reached 70°C by using a thermometer inserted in the central part of the muscle. The cooked samples were then placed under running water to cool them to room temperature. They were blotted dry and weighed once again (W2). Water loss was calculated as the percentage of the initial weight (27).


$$\begin{array}{l} \text{Cooking loss }\left(\text{\%}\right)=\left(\text{W}1-\text{W}2\right)/\text{W}1\times \;100\text{\%} \end{array}$$

### 2.8 Dripping loss measurement

By adopting the natural evaporation method, the muscle was initially weighed (W1). Then it was enclosed in a self-sealed bag to make sure that the bag did not adhere to the meat. After 24 h in the refrigerator at 4°C, the samples were removed and reweighed (W2) (27, 28). The dripping loss was calculated as follows:


$$\begin{array}{l} \text{Dripping loss }\left(\text{\%}\right)=\left(\text{W}1-\text{W}2\right)/\text{W}1\times \;100\text{\%} \end{array}$$

### 2.9 Shearing force measurement

Fresh chicken breasts were collected, and external fat and connective tissue were removed. The samples were then cut into 5 × 2 × 2 cm cubes, and the muscle fibers were gauged using a digital-explicit muscle tenderness meter (C-LM3B, Tenovo International Co., Ltd., Beijing, China). Each sample was measured five times, and the average was acquired (29, 30).

### 2.10 Relative weight of organs

After dissection, the liver, spleen, heart, kidneys and bursa of Fabricius were removed and weighed, and the relative weight was expressed as a percentage to the individual BW (31).

### 2.11 Determination of crude protein content

One gram of muscle was taken, weighted (m), and, placed in a digestion tube. Copper and potassium sulfate were added as catalysts, and sulfuric acid was also added. The muscle was digested using the graphite digestion apparatus (SH220F, Shandong Haineng Technology). After the digested muscle was cooled to room temperature, a K9840 automatic nitrogen determination analyzer (Shandong Haineng Technology) was employed for detection. The blank sample receiving solution was titrated with 0.1 mol/L hydrochloric acid standard titration solution (c) until the color changed, and the volume of hydrochloric acid used for titration (V1) and the titrated volume of the blank sample after detection (V2) was recorded. The crude protein content of the muscle was calculated (32, 33). The calculation formula is as follows:


$$\begin{array}{l} \text{ Intramuscular crude protein content }\left(\text{\%}\right)\;=\;100\text{\%}\\ \times \;6.25\;\times \;\left(\left(\text{V}1-\text{V}2\;\right)\;\times \text{ c }\times \;0.014\;/\text{m}\right) \end{array}$$

### 2.12 Determination of crude fat content

This Soxhlet extraction protocol followed the method of Hopkins et al. (34). Six gram of muscle was taken and weighed (m), which was disposed of in a freeze dryer (SCIENTZ-10 N/A, Ningbo Xin zhi Biology). The initial weight of the Soxhlet extractor cup (m1) was recorded. It was then wrapped in filter paper and, placed in the extraction cup for repeated extraction and rinsed with petroleum ether (30–60°C). After cooling to room temperature, the extraction cup was dried at 105°C and the final weight (m^2^) was recorded. Then the intramuscular fat content was calculated (35). The calculation formula was as follows:


$$\begin{array}{l} \text{Intramuscular fat content }\left(\text{\%}\right)=100\text{\%}\times \;\left(\text{m}1-\text{m}2\right)/\text{m} \end{array}$$

### 2.13 Quantitative real-time polymerase chain reaction (RT-PCR)

Total RNA was extracted from the muscles by means of TRIzol reagent, in accordance with the manufacturer's protocol. The concentrations of extracted RNA were measured by employing a Nanodrop 2,000 spectrophotometer (Thermo Fisher Scientific Inc., Ottawa, ON, Canada). Complementary DNA (cDNA) was generated from RNA with the assistance of an iScriptTM cDNA synthesis kit (Hunan Aikerui Biological Engineering Co., Ltd, Changsha, China). Quantitative real-time polymerase chain reaction (RT-PCR) of genes was carried out using the NCBI primer tool (https://www.ncbi.nlm.nih.gov/). The genes included *IGF1, IGF2, Myod1, Myf5, Myf6, MyoG*, and β*-actin*. Table 2 shows the primers used in this study, which were developed by Shengong Biological Engineering Co., Ltd (Shanghai, China). The cycling conditions were 95°C for 30 s, 40 cycles at 95°C for 5 s, and 60°C for 30 s. Relative gene expression was calculated by means of the 2^−ΔΔ*CT*^ method (36).

**Table 2:** Primer sequences for PCR analysis.

**Gene**	**Primer sequences (5'-3')**	**Accession number**
*β-actin*	F: CAACACAGTGCTGTCTGGTGGTA R: ATCGTACTCCTGCTTGCTGATCC	NM_205518.2
*IGF-1*	F: CCTTGGCCTGTGTTTGCTTAC R: ATCAACCAGCTCAGCACCAC	NM_001004384.3
*IGF-2*	F: CCTTGGCCTGTGTTTGCTTAC R: GCCTCTGTCTCCACATACGA	NM_001030342.5
*Myod1*	F: AAGAGGAAGACCACCAACGC R: GGCTCTCGATGTAGCGGATG	NM_204214.3
*Myf5*	F: CACTTTGTCTCGGGTCGCTA R: AAATCCTCAGGGAACTCGCC	NM_001030363.2
*Myf6*	F: TCGAAACCGGCTCCTATTTCTT R: GTTGGTCCTGACACGGGGAC	NM_001030746.3
*MyoG*	F: GCTCTCTGAGCTGGAAACGG R: AGTGGGAAAGGATTTGGGCG	NM_204184.2

IGF1, Insulin-like growth factor 1; IGF2, Insulin-like growth factor 2; Myod1, myogenic differentiation factor 1; Myf5, myogenic factor 5; Myf6, myogenic factor 6; MyoG, myogenin.

### 2.14 Statistical analysis

SPSS version 26.0 (IBM SPSS, Chicago, IL, USA) and GraphPad Prism version 9.0 (GraphPad Software, San Diego, USA) were used for statistical analysis. Homogeneity of variance test and one-way ANOVA were carried out for data conforming to normal distribution; the LSD method was utilized for the *post-test* of neat variance, and the Tamheni T2 was adopted for irregularity. The test level where *P* < 0.05 was considered to be significant, and *P* < 0.01 was referred to highly significant. All values were presented as mean ± SE.

## 3 Results

### 3.1 Effect of ANE on growth performance in broilers

As outlined in Table 3, Broilers fed diets supplemented with 100, 200, and 400 mg/kg ANE exhibited significant increases in body weight of 15.7%, 15.9%, and 15.0%, respectively, compared to the control group at 21 days of age (*P* ≤ 0.002). In comparison to the control group, all treatment groups, with the exception of the 150 mg/kg ANE group, enhanced the ADG during 1–21 days, especially 100 and 200 mg/kg (*P* < 0.001). All ANE treatment groups lowered the F/G ratio of broilers within 1–21 days (*P* ≤ 0.02). No notable disparities were discernible among all the groups concerning feed intake, average daily weight, and F/G ratio within 22–49 days (*P* > 0.05). However, Dietary supplementation with 200 mg/kg ANE significantly improved both final body weight (9.2% increase) at day 49 and ADG (9.5% increase) throughout the 1–49 day period compared to the control group (*P* ≤ 0.45). These findings demonstrate that dietary ANE supplementation significantly enhanced broiler growth performance, with optimal effects achieved at 100 and 200 mg/kg doses.

**Table 3:** Effects of different levels of ANE on growth performance of AA broilers.

**Items**	**Groups**								**SEM**	**P-value**
	**CON**	**ANE-100**	**ANE-150**	**ANE-200**	**ANE-250**	**ANE-300**	**ANE-350**	**ANE-400**		
**BW, g**										
Initial	40.27	42.11	41.29	41.58	39.85	41.50	40.09	40.85	0.279	0.402
21 d	443.44^b^	513.15^a^	442.30^b^	514.11^a^	487.16^ab^	486.22^ab^	482.94^ab^	510.05^a^	5.708	0.009
49 d	2,297.92^b^	2,342.01^b^	2,358.89^ab^	2,510.68^a^	2,389.82^ab^	2,391.84^ab^	2,329.86^b^	2,237.52^b^	22.034	0.113
**Starter (1–21 d)**										
ADG, g/d	19.20^c^	22.43^a^	20.02^b^	22.65^a^	21.29^ab^	21.26^ab^	21.32^ab^	22.41^a^	0.153	< 0.001
ADFI, g/d	33.34^b^	35.88^a^	33.11^b^	35.76^a^	33.26^b^	34.20^ab^	35.12^a^	34.85^a^	0.168	< 0.001
F/G	1.74^a^	1.60^bc^	1.65^ab^	1.58^c^	1.56^c^	1.61^bc^	1.65^ab^	1.56^c^	0.008	< 0.001
**Grower (21–49 d)**										
ADG, g/d	66.505^a^	65.38^ab^	68.68^a^	71.62^a^	67.93^ab^	67.99^ab^	66.31^ab^	63.61^b^	0.438	< 0.001
ADFI, g/d	111.88^a^	116.79^a^	111.74^a^	117.27^a^	115.48^a^	108.70^a^	117.38^a^	112.96^a^	1.118	0.428
F/G	1.69^ab^	1.79^a^	1.63^a^	1.62^b^	1.70^ab^	1.60^b^	1.76^a^	1.77^a^	0.010	< 0.001
**Overall phase (1–49 d)**										
ADG, g/d	46.23^b^	46.98^ab^	47.82^ab^	50.64^a^	47.94^ab^	47.97^ab^	47.03^ab^	45.95^b^	0.281	< 0.001
ADFI, g/d	78.22^a^	82.11^a^	78.04^a^	82.34^a^	80.24^a^	76.77^a^	82.12^a^	79.48^a^	0.677	0.272
F/G	1.69^ab^	1.75^a^	1.63^ab^	1.62^b^	1.67^ab^	1.60^c^	1.74^a^	1.73^a^	0.012	< 0.001

CON, basal diet supplemented with ANE 0 mg/kg diet; ANE-100, basal diet supplemented with ANE 100 mg/kg diet; ANE-150, basal diet supplemented with ANE 150 mg/kg diet; ANE-200, basal diet supplemented with ANE 200 mg/kg diet; ANE-250, basal diet supplemented with ANE 250 mg/kg diet; ANE-300, basal diet supplemented with ANE 300 mg/kg diet; ANE-350, basal diet supplemented with ANE 350 mg/kg diet; ANE-400, basal diet supplemented with ANE 400 mg/kg diet. BW, body weight; ADG, average daily gain; ADFI, average daily feed intake; F/G, feed-to-weight ratio.

^a, b, c^Means within a row with different superscripts differ significantly (P < 0.05).

### 3.2 Effect of ANE on serum biochemical indices in broilers

As illustrated in Table 4, there were no significant disparities among all ANE- supplemented groups in the levels of ALT, AST, TP, ALB, TG, TC, CREA, HDL-C, LDL-C when compared to the control group (*P* > 0.05). In contrast to the control group, UREA contents were lower in the 200, 300, and 350 mg/kg ANE addition groups (*P* ≤ 0.035). UA activity was increased in the 400 mg/kg ANE addition group as opposed to the control group (*P* = 0.008).

**Table 4:** Effects of different levels of ANE on serum biochemical indicators in Arbor Acres broilers.

**Parameters**	**Groups**								**SEM**	**P-value**
	**CON**	**ANE-100**	**ANE-150**	**ANE-200**	**ANE-250**	**ANE-300**	**ANE-350**	**ANE-400**		
ALT (U/L)	4.14	4.62	3.79	3.68	2.88	2.98	3.05	5.38	0.190	0.712
AST (U/L)	441.52	512.21	446.61	485.7	415.32	418.55	358.09	496.06	16.004	0.443
TP (g/L)	34.72	37.04	30.49	35.20	30.03	28.23	28.63	40.60	0.855	0.481
ALB (g/L)	14.35	15.76	11.95	14.45	11.34	11.04	11.05	16.18	0.408	0.144
TG (mmol/L)	0.53	0.48	0.44	0.49	0.34	0.39	0.40	0.62	0.018	0.729
TC (mmol/L)	5.32	5.73	4.74	5.11	4.18	4.14	4.36	6.57	0.136	0.737
CREA-S (mmol/L)	12.46	7.06	7.08	7.84	7.05	5.26	5.81	4.51	0.508	0.012
UREA (mmol/L)	0.75^a^	0.79^a^	0.68^a^	0.40^b^	0.46^b^	0.45^b^	0.42^b^	0.68^a^	0.025	< 0.001
UA (umol/L)	254.14^b^	291.34^b^	289.17^b^	342.1^ab^	288.69^b^	274.25^b^	271.29^b^	391.16^a^	11.920	0.210
HDL-C (mmol/L)	1,201.4	1,396.18	1,300.28	1,318.53	1,077.67	1,116.33	1,131.90	1,571.43	31.966	0.535
LDL-C (mmol/L)	217.17	268.36	233.12	227.78	175.5	175.50	189.07	279.96	6.630	0.527

CON, basal diet supplemented with ANE 0 mg/kg diet; ANE-100, basal diet supplemented with ANE 100 mg/kg diet; ANE-150, basal diet supplemented with ANE 150 mg/kg diet; ANE-200, basal diet supplemented with ANE 200 mg/kg diet; ANE-250, basal diet supplemented with ANE 250 mg/kg diet; ANE-300, basal diet supplemented with ANE 300 mg/kg diet; ANE-350, basal diet supplemented with ANE 350 mg/kg diet; ANE-400, basal diet supplemented with ANE 400 mg/kg diet. ALT, alanine Aminotransferase; AST, aspartate aminotransferase; TP, total protein; ALB, albumin; TG, triglycerides; TC, total cholesterol; CREA, creatinine; UA, uric acid; HDL-C, high-density lipoprotein cholesterol; LDL-C, low-density lipoprotein cholesterol.

^a, b^Means within a row with different superscripts differ significantly (P < 0.05).

### 3.3 Effect of ANE on the relative organ weights of broilers

Table 5 presents the relative organ weights of broilers following dietary supplementation with ANE. The relative liver weights of all ANE-added groups were lower than those of the control group, among which the 150, 200, and 400 mg/kg ANE added-groups decreased significantly (*P* ≤ 0.046). Additionally, in comparison with the control group, the relative weight of the bursa of Fabricius in the 350 mg/kg ANE addition group decreased markedly (*P* = 0.04). However, no significant difference were observed among all groups in the relative weights of the heart, spleen or kidneys (*P* > 0.05).

**Table 5:** Effects of different levels of ANE on the relative weight of organs in Arbor Acres broilers.

**Relative weight (% of BW of broilers)**	**Groups**								**SEM**	**P-value**
	**CON**	**ANE-100**	**ANE-150**	**ANE-200**	**ANE-250**	**ANE-300**	**ANE-350**	**ANE-400**		
Bursa of fabricius	0.23^a^	0.20^a^	0.17^a^	0.19^a^	0.16^ab^	0.18^ab^	0.11^b^	0.20^a^	0.010	0.223
Cardiac	0.44	0.43	0.45	0.42	0.42	0.47	0.41	0.45	0.010	0.386
Liver	1.97^a^	1.83^ab^	1.70^b^	1.81^ab^	1.77^b^	1.83^ab^	1.87^ab^	1.77^b^	0.020	0.329
Spleen	0.09	0.09	0.13	0.13	0.13	0.11	0.11	0.10	0.001	0.076
Kidney	0.44	0.47	0.47	0.51	0.56	0.53	0.53	0.50	0.010	0.376

CON, basal diet supplemented with ANE 0 mg/kg diet; ANE-100, basal diet supplemented with ANE 100 mg/kg diet; ANE-150, basal diet supplemented with ANE 150 mg/kg diet; ANE-200, basal diet supplemented with ANE 200 mg/kg diet; ANE-250, basal diet supplemented with ANE 250 mg/kg diet; ANE-300, basal diet supplemented with ANE 300 mg/kg diet; ANE-350, basal diet supplemented with ANE 350 mg/kg diet; ANE-400, basal diet supplemented with ANE 400 mg/kg diet. ^a, b^Means within a row with different superscripts differ significantly (P < 0.05).

### 3.4 Effect of ANE on slaughtering performance of broilers

As depicted in Table 6, there were no significant differences among all groups in slaughter rate, half-eviscerated rate, full-eviscerated rate, pectoral muscle rate, leg muscle rate, abdominal fat rate, skin and subcutaneous fat rate (*P* > 0.05), indicating that the addition of ANE in the diet had no significant impact on the slaughter performance of broilers.

**Table 6:** Effects of different levels of ANE on slaughter performance in Arbor Acres broilers.

**Parameters**	**Groups**								**SEM**	**P-value**
	**CON**	**ANE-100**	**ANE-150**	**ANE-200**	**ANE-250**	**ANE-300**	**ANE-350**	**ANE-400**		
Live body weight (g)	2,215.24	2,099.06	2,121.01	2,268.99	2,089.76	2,217.69	2,099.79	2,067.50	26.541	0.594
Slaughter rate (%)	93.63	93.46	93.22	92.16	92.49	93.09	91.94	92.59	0.001	0.042
Half eviscerated rate (%)	88.39	88.39	89.02	88.25	87.94	88.26	87.31	88.21	0.002	0.223
Full eviscerated rate (%)	76.85	76.27	77.51	76.82	76.63	76.84	75.82	76.52	0.002	0.200
Pectoral muscle rate (%)	27.91	29.12	28.71	27.22	28.04	27.35	28.93	28.20	0.004	0.886
Leg muscle rate (%)	24.05	24.42	23.41	23.91	23.46	25.15	24.17	24.06	0.002	0.065
Abdominal fat rate (%)	1.70	1.19	1.69	1.47	1.22	1.19	1.43	1.39	< 0.001	0.231
Skin and subcutaneous fat rate (%)	0.067	0.063	0.070	0.085	0.077	0.069	0.070	0.072	0.001	0.006

CON, basal diet supplemented with ANE 0 mg/kg diet; ANE-100, basal diet supplemented with ANE 100 mg/kg diet; ANE-150, basal diet supplemented with ANE 150 mg/kg diet; ANE-200, basal diet supplemented with ANE 200 mg/kg diet; ANE-250, basal diet supplemented with ANE 250 mg/kg diet; ANE-300, basal diet supplemented with ANE 300 mg/kg diet; ANE-350, basal diet supplemented with ANE 350 mg/kg diet; ANE-400, basal diet supplemented with ANE 400 mg/kg diet.

### 3.5 Effect of ANE on meat quality of broilers

In Table 7, in contrast to the control group, the pectoral muscle pH_45min_ was found to be distinctly elevated in each ANE dosage group, reaching their highest at a dietary concentration of 200 mg/kg ANE (*P* ≤ 0.023), while the pH_24h_ values in all groups were observed to decrease, yet these differences were not found to be significant (*P* > 0.05). The drip loss rate and cooking loss rate of the pectoral muscle presented no significant discrepancy compared to the control group (*P* > 0.05). In comparison with the control group, as the ANE dosage increased, the meat color L^*^ gradually rose, and the meat color a^*^ progressively decreased. Additionally, the b^*^ values of the 250, 300, and 400 mg/kg ANE addition groups were significantly lower than those of the control group (*P* ≤ 0.034). Compared to the control group, the pectoral muscle shear force in the 100, 150, 300, and 350 mg/kg ANE addition groups significantly enhanced (*P* ≤ 0.043).

**Table 7:** Effects of different levels of ANE on the pectoral muscle quality in Arbor Acres broilers.

**Trait (of the pectoral muscle quality)**	**Groups**								**SEM**	**P-value**
	**CON**	**ANE-100**	**ANE-150**	**ANE-200**	**ANE-250**	**ANE-300**	**ANE-350**	**ANE-400**		
PH45min	6.24^c^	6.53^ab^	6.55^ab^	6.66^a^	6.51^ab^	6.49^b^	6.60^a^	6.43^b^	0.020	< 0.001
PH24h	5.94	5.95	5.84	5.87	5.95	5.89	5.82	5.90	0.020	0.407
**Meat color**										
L^*^	49.84	48.49	49.33	51.61	50.50	50.80	51.14	51.08	0.236	0.019
a^*^	6.52	6.36	6.43	6.03	5.97	5.98	5.85	6.05	0.092	0.700
b^*^	11.35^a^	11.10^ab^	11.34^ab^	11.26^ab^	10.35^bc^	9.91^ac^	10.45^abc^	10.31^c^	0.120	0.010
**Water holding**										
Drip loss rate (%)	3.09	3.18	1.97	2.13	2.42	1.96	1.67	1.67	0.001	0.377
Cooking loss rate (%)	30.38	27.78	31.96	31.52	33.23	28.95	30.01	33.85	0.003	0.143
Shear force (N)	33.65^b^	46.02^a^	43.66^a^	34.11^b^	42.44^ab^	44.12^a^	50.27^a^	37.25^b^	1.890	0.048

CON, basal diet supplemented with ANE 0 mg/kg diet; ANE-100, basal diet supplemented with ANE 100 mg/kg diet; ANE-150, basal diet supplemented with ANE 150 mg/kg diet; ANE-200, basal diet supplemented with ANE 200 mg/kg diet; ANE-250, basal diet supplemented with ANE 250 mg/kg diet; ANE-300, basal diet supplemented with ANE 300 mg/kg diet; ANE-350, basal diet supplemented with ANE 350 mg/kg diet; ANE-400, basal diet supplemented with ANE 400 mg/kg diet. L^*^: lightness; a^*^: redness; b^*^: yellowness.

^a, b, c^Means within a row with different superscripts differ significantly (P < 0.05).

As shown in Table 8, the pH_45min_ and pH_24h_ values of the leg muscle in the 200 and 400 mg/kg ANE supplementation groups were remarkably higher than those of the control group (*P* ≤ 0.01). The drip loss and cooking loss exhibited no remarkable differences between the ANE addition groups and the control group (*P* > 0.05). In contrast to the control group, the meat color L^*^ in the 200, 250, and 300 mg/kg ANE addition groups were higher, and the meat color b^*^ in the 400 mg/kg ANE addition group was considerably lower (*P* ≤ 0.05). There was no notable difference in the a^*^ values between the ANE treatment groups and the control group (*P* > 0.05). Additionally, the shear force of leg muscles showed no significant discrepancy in the ANE addition groups in comparison to the control group (*P* > 0.05), except that the 150 mg/kg group decreased conspicuously. These findings suggested that dietary ANE supplementation significantly improved pH and shear force values, ultimately enhancing meat quality parameters.

**Table 8:** Effects of different levels of ANE on the muscle quality of Arbor Acres broiler legs.

**Trait (of the Leg muscle quality)**	**Groups**								**SEM**	***P*-value**
	**CON**	**ANE-100**	**ANE-150**	**ANE-200**	**ANE-250**	**ANE-300**	**ANE-350**	**ANE-400**		
PH_45min_	6.17^b^	6.22^b^	6.36^ab^	6.48^a^	6.32^ab^	6.33^ab^	6.39^a^	6.40^a^	0.020	0.025
PH_24h_	6.03^b^	6.08^b^	6.10^b^	6.25^a^	6.19^ab^	6.19^ab^	6.16^ab^	6.27^a^	0.020	0.100
L^*^	54.92^b^	55.09^b^	54.88^b^	55.61^ab^	57.04^a^	57.22^a^	57.37^a^	55.74^ab^	0.248	0.059
a^*^	8.83	8.33	9.02	8.71	8.98	8.03	8.21	8.44	0.135	0.565
b^*^	13.20	12.25	13.02	13.43	13.12	12.70	12.47	11.24	0.150	0.189
Drip loss rate (%)	2.10	2.02	2.55	1.37	2.48	2.15	1.19	1.85	0.130	0.049
Cooking loss rate (%)	34.40	32.36	37.26	36.32	38.08	30.70	32.79	36.47	0.005	0.452
Shear force (N)	31.49^a^	30.95^ab^	17.94^b^	31.17^a^	28.33^ab^	29.76^ab^	32.18^a^	27.56^ab^	2.276	0.033

CON, basal diet supplemented with ANE 0 mg/kg diet; ANE-100, basal diet supplemented with ANE 100 mg/kg diet; ANE-150, basal diet supplemented with ANE 150 mg/kg diet; ANE-200, basal diet supplemented with ANE 200 mg/kg diet; ANE-250, basal diet supplemented with ANE 250 mg/kg diet; ANE-300, basal diet supplemented with ANE 300 mg/kg diet; ANE-350, basal diet supplemented with ANE 350 mg/kg diet; ANE-400, basal diet supplemented with ANE 400 mg/kg diet. L^*^: lightness; a^*^: redness; b^*^: yellowness.

^a, b^Means within a row with different superscripts differ significantly (P < 0.05).

### 3.6 Effect of ANE on nutrient composition in muscle of broilers

Table 9 shows the effect of ANE on nutrient composition in muscle of broilers, when compared with the control group, the crude fat content in the pectoral muscle of broilers markedly rose after the addition of ANE (*P* ≤ 0.001), among which the 100 mg/kg group had the largest increase extent, approaching approximately 2.8 times that of the control group, while the crude fat content in the leg muscle exhibited a significant increase in the 150 and 350 mg/kg ANE addition groups.

**Table 9:** Effects of different levels of ANE on the nutrient composition in the muscle of Arbor Acres broilers.

**Trait**	**Groups**								**SEM**	***P*-value**
	**CON**	**ANE-100**	**ANE-150**	**ANE-200**	**ANE-250**	**ANE-300**	**ANE-350**	**ANE-400**		
**The content of the pectoral muscle (%)**										
Crude protein (%)	21.10^b^	23.11^a^	22.84^a^	23.25^a^	22.88^a^	21.85^b^	22.01^ab^	22.10^ab^	0.190	0.047
Crude fat (%)	1.00^c^	2.81^a^	2.22^b^	2.24^b^	2.21^b^	2.12^b^	1.97^b^	2.70^ab^	0.093	< 0.001
**The content of the leg muscle (%)**										
Crude protein (%)	20.52^d^	22.14^c^	23.56^b^	24.04^a^	21.37^cd^	23.84^b^	23.18^bc^	22.33^c^	0.231	< 0.001
Crude fat (%)	6.43^b^	7.15^b^	9.58^a^	7.95^b^	6.90^b^	7.88^b^	9.20^a^	7.84^b^	0.322	0.038

CON, basal diet supplemented with ANE 0 mg/kg diet; ANE-100, basal diet supplemented with ANE 100 mg/kg diet; ANE-150, basal diet supplemented with ANE 150 mg/kg diet; ANE-200, basal diet supplemented with ANE 200 mg/kg diet; ANE-250, basal diet supplemented with ANE 250 mg/kg diet; ANE-300, basal diet supplemented with ANE 300 mg/kg diet; ANE-350, basal diet supplemented with ANE 350 mg/kg diet; ANE-400, basal diet supplemented with ANE 400 mg/kg diet.

^a, b, c, d^Means within a row with different superscripts differ significantly (P < 0.05).

Furthermore, the crude protein contents in the pectoral muscles of broilers were enhanced in the 100, 150, 200, and 250 mg/kg ANE supplementation groups as compared to the control group (*P* ≤ 0.018). Except for the 250 mg/kg ANE addition group, the crude protein contents in the leg muscles were elevated in all other testing groups in contrast to the control, especially 200 mg/kg (*P* ≤ 0.036). The results demonstrate that ANE supplementation significantly increased intramuscular nutrient content, thereby improving meat quality parameters.

### 3.7 Effect of ANE on growth and development-related genes in broilers

As the expression results of genes related to muscle development (Table 10) demonstrated, in the pectoral muscle, the level of IGF-1 gene was higher in the 100 and 300 mg/kg ANE addition groups in comparison with the control group (*P* ≤ 0.032), and the expressions of gene IGF-2 in the group with the addition of 200 and 300 mg/kg ANE and the Myod1 in the 100 mg/kg ANE addition group were markedly enhanced compared with the control group (*P* ≤ 0.002). Nevertheless, the level of the Myf5 gene was considerably elevated in all the ANE supplementation groups as against the control group (*P* ≤ 0.001), and the expression of the Myf6 gene was prominently declined in the 200 and 300 mg/kg ANE addition groups (*P* ≤ 0.012); while there was no significant discrepancy in the levels of MYOG gene in the ANE addition groups in comparison to the control group (*P* > 0.05). Furthermore, in the leg muscles of broilers, there were no notable differences in the levels of IGF-1, Myod1, and Myf5 between the ANE treatment groups and the control group (*P* > 0.05). However, the expression of the IGF-2 gene in the 100 and 300 mg/kg ANE supplementation group was significantly increased as opposed to the control group (*P* ≤ 0.032); the levels of the Myf6 gene in the 100, 200, and 300 mg/kg ANE addition group and MYOG in the 400 mg/kg ANE supplementation group were markedly enhanced compared to the control group (*P* ≤ 0.017). Those findings indicated that ANE could promote the muscle growth and development of AA broilers. Notably, lower ANE doses demonstrated superior efficacy in the measured parameters.

**Table 10:** Effects of different levels of ANE on the mRNA relative expression of growth and development related genes in the muscles.

**Parameters**	**Groups**					**SEM**	**P-value**
	**CON**	**ANE-100**	**ANE-200**	**ANE-300**	**ANE-400**		
**The mRNA relative expression of the pectoral muscle**							
*IGF-1*	1.00^b^	1.26^ab^	1.21^ab^	1.35^a^	1.06^b^	0.040	0.027
*IGF-2*	1.00^bc^	1.13^b^	1.54^a^	1.49^a^	0.75^c^	0.069	< 0.001
*Myod1*	1.00^b^	2.17^a^	1.47^b^	1.53^b^	1.33^b^	0.108	0.002
*Myf5*	1.00^c^	1.41^b^	1.75^a^	1.78^a^	1.37^b^	0.059	< 0.001
*Myf6*	1.00^a^	0.99^a^	0.63^b^	0.59^b^	1.18^a^	0.058	< 0.001
*Myog*	1.00	1.18	1.00	0.69	0.83	0.099	0.617
**The mRNA relative expression of the leg muscle**							
*IGF-1*	1.00^ab^	0.77^b^	1.04^ab^	1.21^a^	0.89^b^	0.042	0.012
*IGF-2*	1.00^c^	1.76^a^	1.10^bc^	1.27^b^	0.87^c^	0.067	< 0.001
*Myod1*	1.00	1.20	1.25	1.12	1.05	0.060	0.705
*Myf5*	1.00^ab^	1.02^ab^	1.12^a^	0.91^b^	1.11^ab^	0.032	0.211
*Myf6*	1.00^b^	1.60^a^	1.73^a^	1.56^ab^	1.11^b^	0.083	0.008
*Myog*	1.00^b^	0.82^b^	0.80^b^	1.05^b^	1.44^a^	0.061	0.002

CON, basal diet supplemented with ANE 0 mg/kg diet; ANE-100, basal diet supplemented with ANE 100 mg/kg diet; ANE-150, basal diet supplemented with ANE 150 mg/kg diet; ANE-200, basal diet supplemented with ANE 200 mg/kg diet; ANE-250, basal diet supplemented with ANE 250 mg/kg diet; ANE-300, basal diet supplemented with ANE 300 mg/kg diet; ANE-350, basal diet supplemented with ANE 350 mg/kg diet; ANE-400, basal diet supplemented with ANE 400 mg/kg diet.

^a, b, c^Means within a row with different superscripts differ significantly (P < 0.05).

## 4 Discussion

Since the ban on antibiotics, poultry diseases have gradually occurred, severely affecting animal welfare. As the consumption of processed products has increased dramatically, consumers are more attentive to quality of meat (37). It is noteworthy that the decline in animal welfare can have a negative impact on poultry growth performance and meat quality. AN, a natural plant with a 2,000-year history of edible and medicinal use, contains valuable bioactive constituents such as alkaloids, polyphenols, polysaccharides, and fatty acids. It has been conclusively demonstrated to possess a repertoire of bioactive functions, including anti-inflammatory, antioxidant, and anti-parasitic activities, etc. (38), thereby emerging as a potential feed additive in poultry nutrition. To determine the optimal dosage of ANE that could simultaneously enhance animal growth and improve meat quality, a series of experimental groups were established. Each group consisted of 16 chickens housed and fed in 3 cages. Such an experimental design not only meets statistical significance requirements, but also complies with the requirements and regulations of the Animal Welfare Act. The augmented body weight of broilers supplemented with ANE in the present study accords with the findings of Wang (15), who reported that ANE treatment significantly enhanced the growth and decreased the FCR in Wenchang chickens. Additionally, we noted that in the early growth stage of broilers, in comparison to the control group, the ADG rose by 16.8% and 18%, respectively, when 100 and 200 mg/kg ANE were added to the diet. The 18.2% ADG improvement in the 200 mg/kg ANE group exceeds reported effects of oregano essential oil (12–15% ADG gain) (39) and aligns with bacitracin methylene disalicylate (20–22% gain) (40). However, based on the outcome yielded in this investigation, there was no significant impact on the ADFI, which is consistent with Lee's findings (41). Previous studies have demonstrated that arecoline can stimulate the sympathetic nervous system, boost salivary secretion and gastrointestinal peristalsis, and assist in improving digestive function (12). Our study revealed that higher weight of AA broilers at the age of 1–21 days was achieved with ANE supplementation, resulting in a remarkedly lower F/G ratio. The reason for this alteration might be that the ANE possesses the function of promoting digestion (7), indicating that ANE has potential as a feed additive. Poultry meat production performance is mainly evaluated by its slaughter rate and eviscerating rate (42). The superior meat quality is mainly manifested in that the slaughter rate exceeds 80%, and the eviscerating rate surpasses 60%. The results of our experiment demonstrated that the supplementation of ANE resulted in a slaughter rate of over 90% and an eviscerating rate exceeding 70% in broilers. These findings suggest that the appropriate supplementation of ANE can effectively improve the meat quality of broilers.

The excessive fat deposition in broilers, particularly the increase of abdominal fat, may be a contributing factor to the declined feed conversion ratio and meat quality (43, 44). The current study indicated that dietary ANE could reduce the abdominal fat rate of broilers to a certain degree, which may be closely related to arecoline, the main component of ANE. This finding is in line with that of Xu et al. (45), who observed the promotion of lipid metabolism in mice. It is manifested by the increased serum TC, TG, and liver TC levels and the reduction of abdominal fat accumulation.

The quality of meat is mainly appraised by gauging its pH value, meat color, drip loss, cooking loss, and shearing force (46). The pH of muscles generally ranges between 7 and 7.5, which is determined by the amount of lactic acid present. Excessive accumulation of lactic acid in muscles leads to a decrease in pH, lowering its quality. Generally, the greater the pH value in the meat, the better the tenderness. A rapid drop in pH will result in paler and less fresh meat (47). Studies have shown that muscle glycogen, pH, and related meat quality traits are both regulated by genetics and nutrition (48). The chicken with thinner pectoral muscles is characterized by a higher pH in the breast (49). At 45 min after slaughter, our study revealed that ANE increased the pH of muscles. Particularly, the pH of pectoral muscles significantly increased in the presence of ANE compared to the control group. At 24 h post-slaughter, the pH of leg muscles remained higher than that of the control group, while there was no alteration in the pectoral muscles. It indicated that the appropriate dose of ANE supplementation guarantees the freshness of broiler meat. Meat color is the most visual indicator of physiological and biochemical changes in muscle. The meat color index is comprised of three values: L^*^, a^*^, and b^*^. These values are influenced by myoglobin and hemoglobin, especially the redox degree of myoglobin (50). The L^*^ value reflects brightness, while the b^*^ value represents the oxidation level of meat fat and the extent of bacterial growth (51). The present findings illustrate that supplementation with ANE results in a notable increase in the L^*^ value of pectoral and leg muscles, thereby enhancing the quality of the meat. This may be related to the flavonoids present in ANE (52). Furthermore, the current study showed that the decreased redness a^*^ and yellowness b^*^ values in comparison with the control group signified that the oxidation of muscle fat was inhibited upon supplementation with ANE, thereby improving meat quality. These results might be associated with the polyphenols present in ANE (53). Additionally, the ethanol extract of ANE has been demonstrated in numerous studies to possess potent antioxidant properties, and the polyphenols epicatechin and syringic acid in ANE play an important role (54). A smaller shearing force indicates greater muscle tenderness, while drip loss and cooking loss reflect the ability of the muscle to retain water in different environments (55, 56). In comparison to the control group, the ANE supplementation group reduced the drip loss of broilers, though no significant distinction was observed. And the addition of ANE resulted in an increase in shearing force observed in the pectoral muscle, while a decrease was moted in the leg muscle. The elevated shearing force values of beef, pork, and chicken are attributed to the presence of fat cells surrounding the muscle fibers (56). However, the shearing force of cooked meat is reduced by the accumulation of fat cells around the muscle (57), and more in-depth study is required to clarify the mechanism.

The relative weights of internal organs, in particular the liver and spleen, are commonly utilized indicators for evaluating immune status and physiological health in poultry (58). In the present study, the relative liver weights were significantly lower in broilers supplemented with ANE compared to the control group. This finding may be related to the antioxidant function of ANE (7). However, ANE supplementation didn't alter the relative weights of other examined organs, including the kidneys and spleen, consistent with the findings by Li et al. (59). This suggests that the supplementation of ANE did not adversely affect the overall normal health and wellbeing of the broilers during the course of the research. In addition, our findings align with Sari et al. (60), who reported that a dose of 1,000 mg/kg oral ANE was the most efficacious in the cellular immune model, and there were no adverse effects on the liver and kidney functions. Nevertheless, further research is needed to ascertain the safety limit of ANE for hepatic and renal functions.

Serum biochemical indicators are crucial indicators for reflecting the material metabolism and functional alterations of certain tissues and organs of animals (61). The high content of TP enhances the metabolism of the body, leading to the growth and development of animals (60). Our current research discovered a higher serum TP in the 100 mg/kg ANE addition group compared to the control group, indicating that protein synthesis can be promoted through ANE supplementation, resulting in a promoting effect on the growth and development of the body. Similar studies showed that the levels of TP and ALB in the blood of broilers were increased by triterpenoids contained in rosemary extract (62). Typically, the lipid metabolism in the body can be reflected by the content of TC in serum. The greater the content of TC in serum, the higher the occurrence of cardiovascular, cerebrovascular and kidney diseases (63). The present study discovered that ANE supplementation in broilers could reduce serum TC, which aligns with Hu et al. (64), who reported that the level of TC can be declined by the plant extract berberine. UA is the end product of purine metabolism, and also a biomarker in kidney disease. Low UA facilitates protein digestion and absorption, stabilizing the body's physiological function (65). Rosebrough et al. (66) found that the plasma UA content of broilers can be augmented by the reduced dietary energy ratio. Our study demonstrated that the level of UA in serum increased significantly with the addition of 400 mg/kg ANE. It signified that catabolism of proteins might be altered, meaning there was a burden on the kidneys of broilers with this dose. Nevertheless, the current study indicated that ANE supplementation induced no significant impact on the serum levels of ALT, AST, CREA and UREA in broilers, indicating the absence of toxic or adverse effects at the administered dose. This finding is consistent with the hepatoprotective properties of ANE on hepatic biomarkers reported by Pithayanukul et al. (67).

As we all know, protein and fat levels are two of the most crucial factors influencing meat quality. Furthermore, they serve as indicative markers of the nutritional profile of meat (68). Moderate levels of fat cannot only enhance the tenderness of meat, but also be related to the juiciness and flavor of meat. Generally, the lower the crude fat (CF) in muscle, the poorer the fat deposition capacity and meat flavor will be (69). Keeping the intramuscular fat content within 2% to 3% can promise the meat flavor to be the best (70). The findings of the present study showed that the intramuscular fat content could be significantly increased by the addition of ANE, especially with the 100 mg/kg ANE supplementation. The intramuscular fat content of the pectoral muscle was observed to reach 2.81%, which was 1.8 times higher than that of the control group. Additionally, the intramuscular fat content of the leg muscle was observed to reach 9.58% with 150 mg/kg ANE supplementation, which was 48.9% higher compared to the control group. The observed increase in intramuscular fat content suggested that the meat flavor of broilers could be improved with ANE supplementation. A previous study (68) has reported that the higher the crude protein in muscle, the greater the content, indicating high nutritional value. The current study revealed that the crude protein content in the pectoral muscle and the leg muscle was significantly increased, which indicated that the muscle quality and nutritional value could be significantly improved with ANE supplementation.

Muscle growth is a dynamic process involving anabolism and catabolism. Insulin-like growth factors (IGFs) are a group of polypeptides that contribute to muscle growth. Most of them are synthesized in the liver and released into the bloodstream to act on target cells, thereby promoting growth and regulating metabolism (71). *IFG-1* is a peptide hormone mainly produced by the liver and stimulated by growth hormone (GH). *IFG-1* increases the total number of cells in muscle with adequate protein, resulting in facilitating the growth of new muscle cells (52). Additionally, *IFG-1* is associated with connective tissue generation and also plays an important role in glucose metabolism along with insulin (72). *IIFG-2* is an embryonic growth factor and a major growth-promoting hormone during pregnancy, which regulates cell proliferation, growth, migration, differentiation, and survival (73). In our study, the mRNA expressions of *IFG-1* and *IFG-2* were conspicuously higher in the pectoral and leg muscles with ANE supplementation, except for the 400 mg/kg ANE addition group, compared to the control group. It indicated that *Igfs* did not play a crucial role with 400 mg/kg ANE supplementation. Davis (74) discovered that myogenic regulatory factors are important regulatory elements in the process of myogenesis. They play a vital role in the development and occurrence of muscles, as well as in guiding the differentiation of muscle satellite cells and skeletal muscle regeneration. Skeletal myogenesis is regulated by four myogenic regulatory factors (Mrfs), including *Myf5, Myod, Myog*, and *Mrf4* (75). Mrfs coordinate multiple periods of the muscle lineage, such as various stages of skeletal muscle development, from myoblast proliferation and cell cycle exit to myoblast fusion and myotube maturation (76). Among them, *Myod1, Myf5, Myog*, and *Myf6* exert a crucial role in regulating muscle growth and development. Our study demonstrated that the mRNA expression of *Myod1* and *Myf5* in pectoral and leg muscles were significantly augmented, suggesting that myogenic cell typing and proliferation were enhanced. Moreover, early skeletal muscle development was facilitated by ANE supplementation. *Myod* primarily assumes an important part in the process of muscle differentiation and is expressed at the end of differentiation. As a transcription activator, it promotes the transcription of muscle-specific target genes. It is also significant for muscle differentiation, cell cycle exit and muscle atrophy (77). *Myf6* transcribes and regulates a broad spectrum of muscle factors and muscle-secreted proteins in skeletal muscle fibers, resulting in the establishment of ligand-receptor interactions between muscle stem cells and their related muscle fibers (78). Nevertheless, the mRNA expression of *Myf6* in pectoral muscles was markedly decreased with 200 and 300 mg/kg ANE supplementation. This indicated that the process of muscle differentiation and maturation was faster at the end of the period. Previous research revealed that two polymorphic loci in the 5′ regulatory region of the *Myog* gene have notable impacts on the muscle fiber density, muscle weight, leg muscle weight and leg muscle development rate of chickens (79). This might be the reason why the *Myog* mRNA expression in the pectoral muscle is contrary to that in the leg muscle in our study. The early transcription factors of *Myod1* and *Myf5* mRNA expression are not significantly higher than that of *Myf6* in leg muscles. This indicated that the early development of leg muscles was more rapid and the mature stage of muscle fibers was more evident. However, it was still shown that ANE could boost the expression of genes related to muscle growth and development, thereby exerting a promoting role in muscle growth and development. It can, therefore, be concluded that ANE exerts a beneficial effect on broilers. The present study demonstrated that ANE promoted growth performance, prevented pH from dropping in muscles, and raised the flesh color value of pectoral muscle, as well as the crude fat and crude protein content in the muscle of broilers.

While the current findings are promising, further investigations are still needed to elucidate the metabolic mechanisms underlying ANE's effects in poultry and identify the primary active constituents responsible for its biological activity. Moreover, it is imperative to expand sample sizes through on-farm trials in commercial poultry faccilities to validate efficacy under real-world production conditions, while concurrently, conducting longitudinal safety assessments to establish a robust foundation for regulatory approval of ANE as a novel feed additive.

## 5 Conclusion

This study discovered that ANE exerted positive effects on AA broilers. An appropriate amount of ANE in the diet has been demonstrated to boost the growth performance and meat quality of broilers, facilitate muscle development, and has no remarkable influence on slaughter performance. These results can provide further evidence using ANE as a feed additive in broiler production. Considering both the experimental results and economic feasibility, it is recommended that the optimal dosage for broilers is 100–200 mg/kg ANE.

## Data Availability

The datasets presented in this study can be found in online repositories. The names of the repository/repositories and accession number(s) can be found in the article/supplementary material.
